# Sodium salicylate modulates inflammatory responses through AMP‐activated protein kinase activation in LPS‐stimulated THP‐1 cells

**DOI:** 10.1002/jcb.26249

**Published:** 2017-08-30

**Authors:** Weiwei Bao, Yaru Luo, Dan Wang, Jian Li, Xi Wu, Wei Mei

**Affiliations:** ^1^ Department of Anesthesiology, Tongji Hospital, Tongji Medical College Huazhong University of Science and Technology Wuhan Hubei China; ^2^ Department of Anesthesiology, Xinqiao Hospital The Third Military Medical University Chongqing China; ^3^ Department of Anesthesiology, Renmin Hospital of Wuhan University Hubei Province Wuhan Hubei China; ^4^ Department of Anesthesiology Shenzhen Second People's Hospital Guangdong Province Shenzhen China

**Keywords:** AMP‐activated protein kinase (AMPK), apoptosis, cytokine, signal transducer and activator of transcription 3 (STAT3), lipopolysaccharide (LPS), proliferation, sodium salicylate (NaSal)

## Abstract

Sodium salicylate (NaSal) is a nonsteroidal anti‐inflammatory drug. The putative mechanisms for NaSal's pharmacologic actions include the inhibition of cyclooxygenases, platelet‐derived thromboxane A2, and NF‐κB signaling. Recent studies demonstrated that salicylate could activate AMP‐activated protein kinase (AMPK), an energy sensor that maintains the balance between ATP production and consumption. The anti‐inflammatory action of AMPK has been reported to be mediated by promoting mitochondrial biogenesis and fatty acid oxidation. However, the exact signals responsible for salicylate‐mediated inflammation through AMPK are not well‐understood. In the current study, we examined the potential effects of NaSal on inflammation‐like responses of THP‐1 monocytes to lipopolysaccharide (LPS) challenge. THP‐1 cells were stimulated with or without 10 ug/mL LPS for 24 h in the presence or absence of 5 mM NaSal. Apoptosis was measured by flow cytometry using Annexin V/PI staining and by Western blotting for the Bcl‐2 anti‐apoptotic protein. Cell proliferation was detected by EdU incorporation and by Western blot analysis for proliferating cell nuclear antigen (PCNA). Secretion of pro‐inflammatory cytokines (TNF‐α, IL‐1β, IL‐6) was determined by enzyme‐linked immunosorbent assay (ELISA). We observed that the activation of AMPK by NaSal was accompanied by induction of apoptosis, inhibition of cell proliferation, and increasing secretion of TNF‐α and IL‐1β. These effects were reversed by Compound C, an inhibitor of AMPK. In addition, NaSal/AMPK activation inhibited LPS‐induced STAT3 phosphorylation, which was reversed by Compound C treatment. We conclude that AMPK activation is important for NaSal‐mediated inflammation by inducing apoptosis, reducing cell proliferation, inhibiting STAT3 activity, and producing TNF‐α and IL‐1β.

## 1 INTRODUCTION

AMPK, a crucial sensor of cellular energy status^1^, ^2^ is composed of a catalytic α subunit and regulatory β and γ subunits.^3^ Activation of AMPK promotes fatty acid oxidation, utilizes catabolic pathway and inhibits aerobic glycolysis to conserve ATP. Activated immune cells and proliferating cells (including tumor cells) tend to utilize aerobic glycolysis, while their inactivated or anti‐inflammatory cells tend to utilize fatty acid oxidation to generate ATP,^4^ supporting the idea that activation of AMPK can have anti‐inflammatory effects. Recently, AMPK has been shown to regulate the skewing of the macrophage from a pro‐ to anti‐inflammatory phenotype at the time of resolution of inflammation.^5^

In agreement with the role of AMPK in suppressing inflammatory responses, AMPK has been proposed to be an inducer of apoptosis and inhibitor of cell proliferation.^6^ In mammals, rapidly proliferating cells tend to utilize aerobic glycolysis to satisfy their high demand for ATP.^4^ Once activated, AMPK inhibited cell growth and cell proliferation by promoting ATP conservation.^7^ In addition, when AMPK is activated, various AMPK‐regulated pathways such as activation of the p53‐p21 axis, inhibition of mTOR signaling, and promotion of fatty acid oxidation all inhibit cell proliferation.^8^ Apoptosis is a highly controlled means of eliminating unwanted or dangerous cells such as immunological tolerance, inflammation without causing an inflammatory response, or tissue damage.^9^, ^10^ The pro‐apoptotic potential of AMPK in AMPK over expressed conditions has also been reported, with AMPK activators or constitutively active AMPK mutants.^11^, ^12^ These suggested that AMPK‐activating drugs might be useful as a therapeutic agent for the treatment of inflammation‐related diseases.

Salicylate, an aspirin metabolite is widely used for the treatment of inflammation‐related diseases.^13^ Putative mechanisms for salicylate pharmacologic actions include inhibition of cyclooxygenases and NF‐κB signaling.^14^, ^15^ Reports have shown that salicylate could activate AMPK directly, causing allosteric activation, and promoting Thr‐172 phosphorylation.^16^ However, so far, little has been reported regarding the role of AMPK activated by salicylate in inflammatory cells. Salicylate also possesses anti‐proliferation and pro‐apoptosis potency in inflammatory conditions.^17^, ^18^ However, the precise mechanism by which salicylate exerts anti‐proliferation and pro‐apoptosis effects during inflammation remains unclear. We address the question whether the anti‐inflammatory effects of salicylate are partly mediated by AMPK and how salicylate modulates inflammation in LPS‐stimulated THP‐1 cells. The monocyte is an important part of the immune system and a key cell type in sepsis. Human monocytes are exquisitely sensitive to LPS and orchestrate the innate immune response to LPS including expression of inflammatory cytokines.^19^ THP‐1 cells have been widely used as a model to study the immune response capacity of monocytes.

In this study, we investigated the potential effects of AMPK in NaSal‐mediated inflammation in LPS‐stimulated THP‐1 monocytes.

## 2 MATERIALS AND METHODS

### 2.1 Reagents

NaSal, 5‐Aminoimidazole‐4‐carboxamide‐1‐b‐ribofuranoside (AICAR), and LPS (Escherichia coli serotype 055. B5) were obtained from Sigma‐Aldrich (St. Louis, MO). Compound C was purchased from Selleckchem (Houston, TX).

### 2.2 Cell culture and treatments

A human monocyte cell line, THP‐1 (obtained from Cell Bank, Wuhan University) was cultured in RPMI‐1640 (Gibco, Beijing, China, Intvitrogen, Cell Signaling Technology, Abbkine, ThermoFisher Scientific, Bio‐Rad, and BD Biosciences) with 10% fetal bovine serum (FBS, Gibco, New Castle, Australia) at 37°C in a humidified incubator in an atmosphere of 5% CO_2_ and 95% air. In all experiments, THP‐1 cells (7 × 10^5^ cells/mL) were seeded in plates and incubated overnight. Cells were pretreated with 1 µM concentration of Compound C for 30 min followed by stimulation with, or without 10 μg/mL LPS in the presence or absence of 5 mM NaSal or 2 mM AICAR for 24 h.

### 2.3 Western blot analysis

Cells were collected, washed twice with cold PBS, and lysed for 30 min on ice in cell extraction buffer (Invitrogen, Camarillo, CA), with vortexing at 10 min intervals. Lysates were then centrifuged at 12 000 rpm for 10 min at 4°C. The protein concentration was measured using a BCA Protein Analysis kit (Boster, Wuhan, China) according to the manufacturer's instructions. Equal amounts of protein (30 µg) were separated by electrophoresis in 10% sodium dodecyl sulfate‐polyacrylamide gels (SDS‐PAGE) and transferred to PVDF membranes. Membranes were soaked in 5% bovine serum albumin (BSA) in PBS and then incubated overnight at 4°C with the following primary antibodies: p‐AMPK‐Thr172 (1:1000; Cell Signaling Technology, Boston, MA), AMPK‐1α (1:1000; Cell Signaling Technology), p‐STAT3‐Tyr705 (1:2000; Cell Signaling Technology), STAT3 (1:2000; Cell Signaling Technology), Bcl‐2 (1:1000; Cell Signaling Technology), PCNA (1:1000, Cell Signaling Technology), or β‐actin(1:500; Boster, Wuhan, China). After thorough washing with TBST, membranes were then incubated with horseradish peroxidase (HRP)‐conjugated goat anti‐mouse or anti‐rabbit IgG antibody (1:5000; Abbkine, Wuhan, China) for 2 h at room temperature. Blots were detected using the enhanced chemiluminescence (ECL) reagents (ThermoFisher Scientific, Rockford, IL). Labeled proteins were detected with a Chemi‐Doc XRS imaging system (Bio‐Rad, Hercules, CA). The relative expression levels of Bcl‐2 and PCNA were normalized to that of the housekeeping gene β‐actin. The relative expression levels of p‐AMPK or p‐STAT3 were normalized to AMPK‐1α or STAT3. Values are expressed as relative optical density. The blot density of control groups was set as 1. All experiments were performed at least three times.

### 2.4 Enzyme‐linked immunosorbent assay (ELISA)

Culture supernatants were collected after treatment and stored at −80°C until assayed for cytokines. Levels of IL‐1β, IL‐6 (ExCell Bio, Shanghai, China), and TNF‐α (R&D Systems, Minneapolis, MN) were measured by ELISA kits according to the manufacturer's instructions. Absorbance was measured at 450 nm using a microplate reader. All samples were performed in triplicate.

### 2.5 Annexin V/PI assay

Apoptotic cells were measured using the BD Pharmingen™ FITC Annexin V Apoptosis Detection Kit (BD Biosciences, San Diego, CA). After treatment for 24 h, cells were washed twice with cold PBS and then resuspended in 1x Binding Buffer. After transferring 100 μL of the solution to a 5 mL culture tube, 5 μL FITC Annexin V, and 5 μL PI were added to the tube. Cells were gently vortexed and then incubated for 15 min at RT(25°C) in the dark. After adding 400 μL 1x Binding Buffer to each tube, the cells were analyzed by FACS. The extent of apoptosis was quantified as the percentage of Annexin V‐positive cells.

### 2.6 EdU incorporation assay

For EdU incorporation experiments, cells were performed as described above. 5‐ethynyl‐2′‐deoxyuridine (EdU) was added at a 10 µM final concentration to the cells 3 h before harvesting. Cell proliferation was detected using the incorporation of EDU with the Click‐iT Plus EdU Flow Cytometry Assay Kits (Invitrogen, Eugene, OR). Briefly, the cells were incubated with 10 µM EdU for 3 h before fixation and permeabilization. All procedures were performed according to the manufacturer's protocol. EdU‐positive cells were determined by flow cytometry on a FACScan.

### 2.7 Statistical analysis

All data are expressed as the mean ± S.E.M. Statistical analysis was performed using the GraphPad Prism version 5.01 for Windows (Graph Pad Software, San Diego, CA). Student's *t*‐test was used for comparison between two samples. Statistical comparisons of more than two groups were performed using one‐way ANOVA followed by the Dunnett test. A *P* value <0.05 was considered to be statistically significant.

## 3 RESULTS

### 3.1 NaSal induces p‐AMPK in LPS‐stimulated THP‐1 monocytes

**Figure 1:**
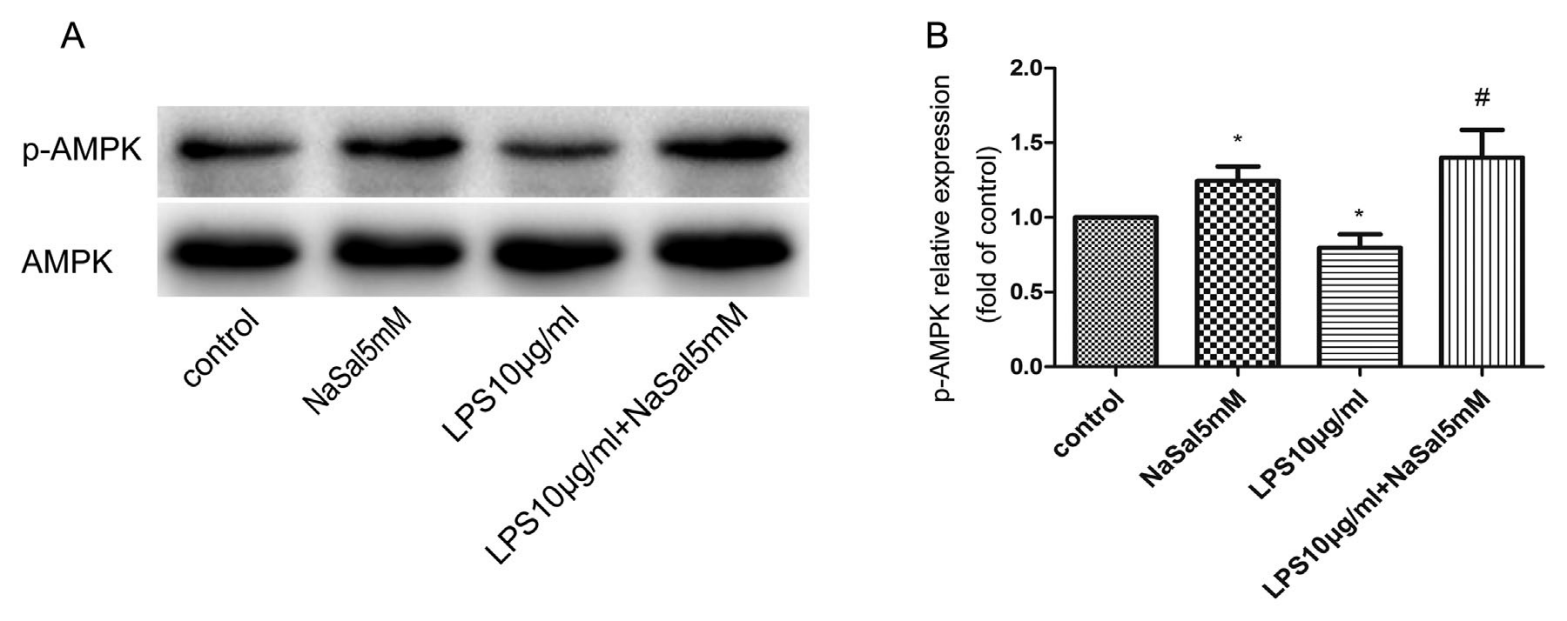
Phosphorylation of AMPK by NaSal in LPS‐stimulated THP‐1 monocytes. THP‐1 cells were treated with LPS (10 μg/mL) in the presence or absence of NaSal (5 mM) for 24 h and measured p‐AMPK (Thr172), AMPK‐1α protein levels by Western blotting. (A,B) p‐AMPK protein expression was assessed by Western blotting and densitometry. Values of p‐AMPK were normalized to those for AMPK1α and are expressed as relative optical density. The relative expression of p‐AMPK in the control group was set at 1 for quantification. The Western blot data (A) is 1 representative experiment, and the graph then represents data from *n* > 3 replicate experiments (B). The data represent the mean ± S.E.M. of *n *> 3. *P < 0.05 compared with the control group. ^#^ *P* < 0.05 compared with the LPS treatment group

We treated LPS‐stimulated THP‐1 cells with NaSal and examined the total and phosphorylation levels of AMPK in different groups by Western blot analysis. As shown in Fig. 1A,B, the expression of p‐AMPK decreased in LPS‐stimulated monocytes, while treatment with NaSal reversed the LPS‐stimulated down‐regulation of p‐AMPK. The total protein level of AMPK shows no obvious changes in different groups.

### 3.2 Effects of NaSal on LPS‐stimulated THP‐1 monocytes apoptosis and cell proliferation

**Figure 2:**
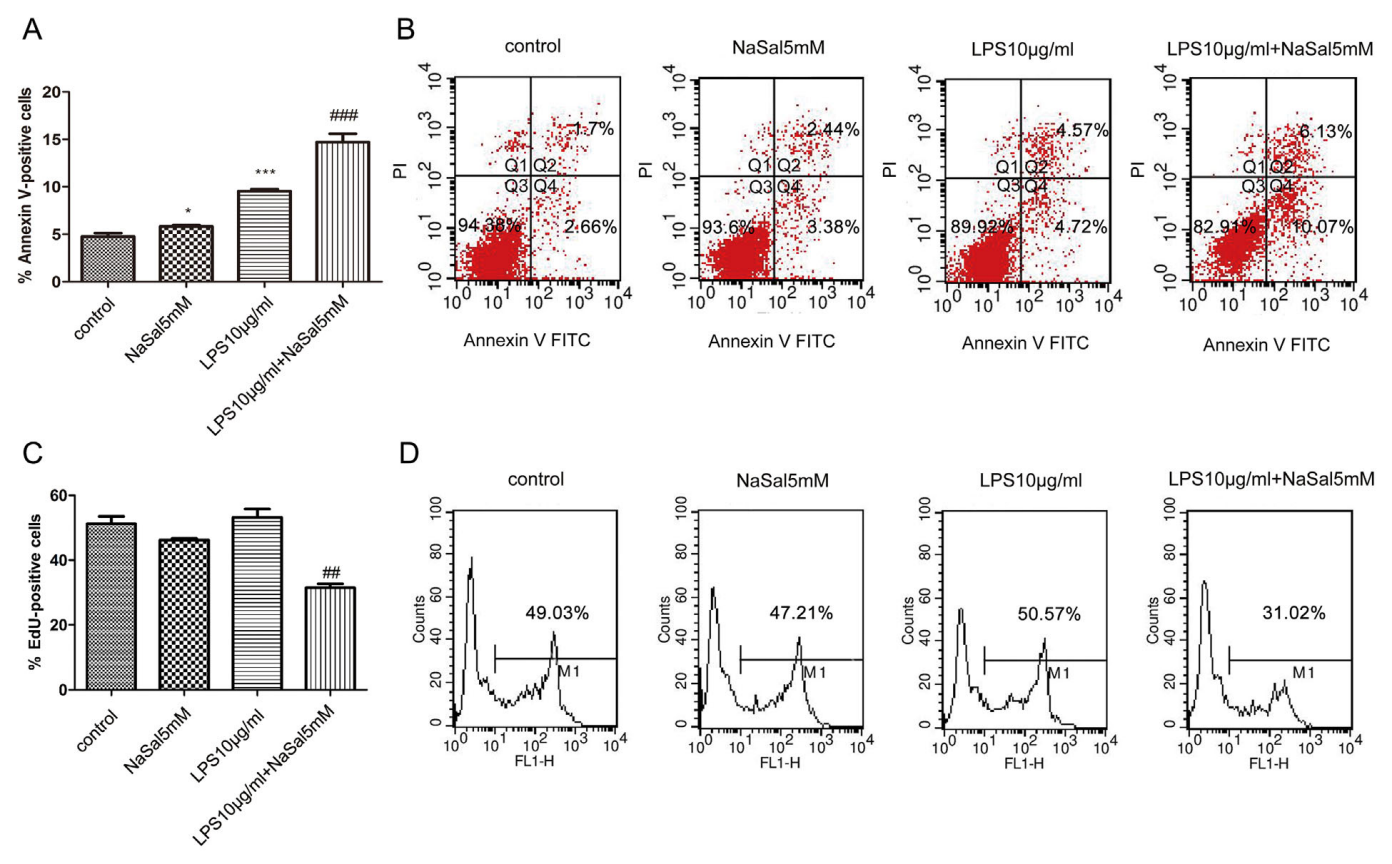
Effects of NaSal on LPS‐stimulated THP‐1 monocytes apoptosis and cell proliferation. THP‐1 cells were treated with LPS (10 μg/mL) in the presence or absence of NaSal (5 mM) for 24 h, and later assayed for apoptosis by flow cytometric analysis using Annexin V/PI staining (A,B), as well as cell proliferation using flow cytometry with anti‐5‐ethynyl‐2′‐deoxyuridine (anti‐EdU) Alexa Fluor 488 staining (C,D). The flow cytometry data (B,D) is 1 representative experiment, and the graph then represents data from *n* = 3‐5 replicate experiments (A,C). The data represent the mean ± S.E.M of *n* = 3‐5. *P < 0.05 and ***P < 0.001 compared with the control group. ^##^ *P* < 0.01 and ^###^ *P* < 0.001 compared with the LPS treatment group

Previous studies have shown that aspirin/salicylate enhances apoptosis and reduces cell proliferation in colorectal cancer,^20^ B‐chronic lymphocytic leukemia (B‐CLL) cells,^21^ vascular smooth muscle cells,^22^ and human pancreatic cancer cell lines.^17^ Next, we examined the effects of NaSal on apoptosis and cell proliferation in LPS‐stimulated THP‐1 cell. Cells were stimulated with 10 μg/mL LPS for 24 h in the presence or absence of 5 mM NaSal. Apoptosis of the THP‐1 cells was analyzed using Annexin V/PI staining. Cell proliferation was detected by EdU incorporation assay using flow cytometry. As shown in Fig. 2A, treatment of NaSal significantly induced cell apoptosis in LPS‐stimulated THP‐1 cells. The percentage of apoptotic cells increased significantly from 9.52 ± 0.23% of the LPS‐stimulated THP‐1 cells to 14.69 ± 0.89% of the THP‐1 cells treated with LPS and NaSal (*P* < 0.001; Fig. 2A,B). As indicated in Fig. 2C, treatment of LPS‐stimulated THP‐1 cells with NaSal led to a significant decrease in cell proliferation. The EdU incorporation rate was reduced to 31.02% (*P *< 0.01, Fig. 2D) in the cells treated with LPS and NaSal compared with the cells stimulated with LPS alone.

### 3.3 AICAR induces p‐AMPK, promotes apoptosis, and inhibits cell proliferation in LPS‐stimulated THP‐1 monocytes

**Figure 3:**
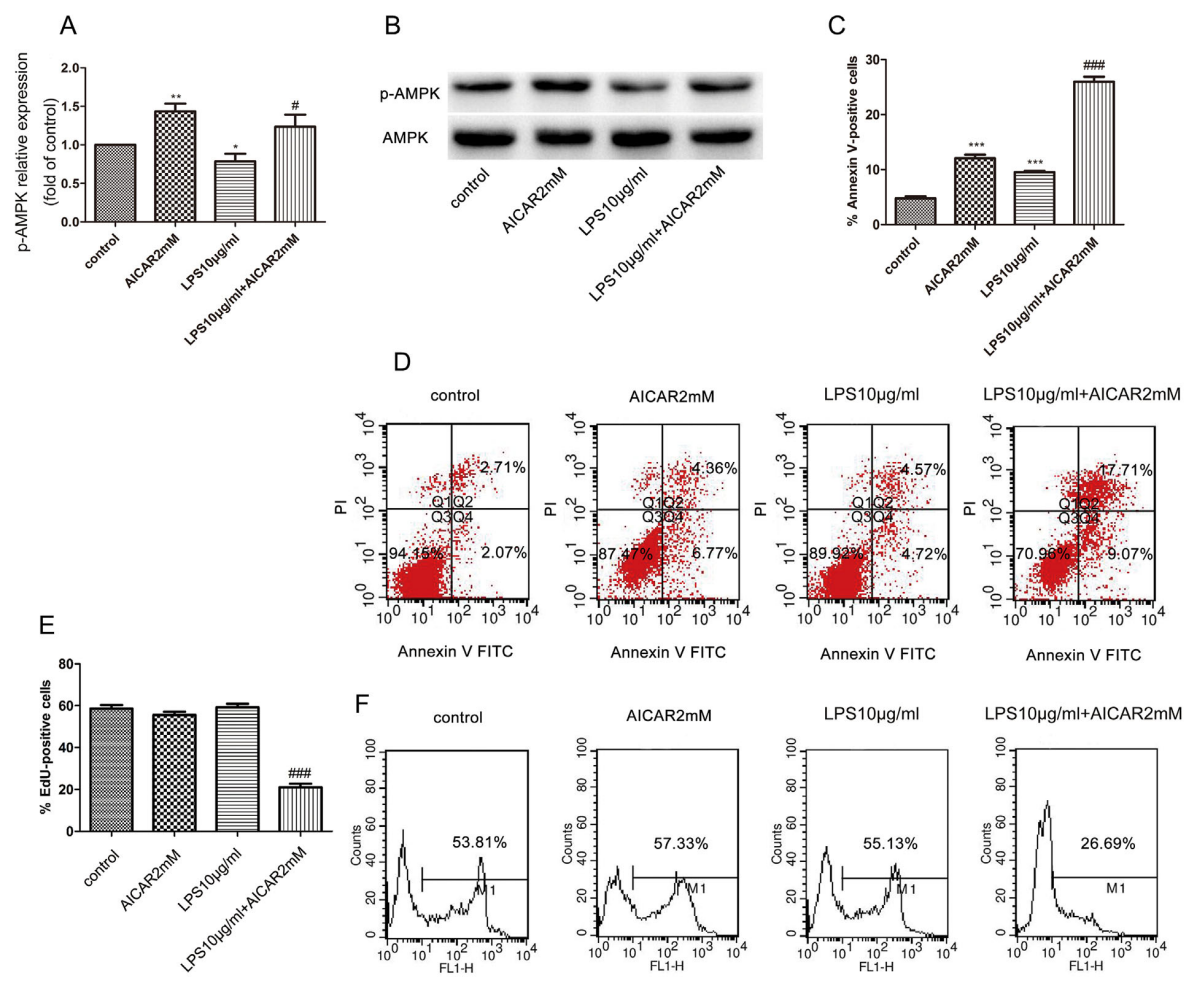
Effects of AICAR on AMPK activation, apoptosis, and cell proliferation in LPS‐stimulated THP‐1 monocytes. THP‐1 cells were treated with LPS (10 μg/mL) in the presence or absence of AICAR (2 mM) for 24 h and measured p‐AMPK (Thr172), AMPK‐1α protein levels by Western blotting (A,B). p‐AMPK protein expression was assessed by Western blotting and densitometry. Values of p‐AMPK were normalized to those for AMPK1α and are expressed as relative optical density. The relative expression of p‐AMPK in the control group was set at 1 for quantification. Apoptosis (C) and cell proliferation (E) were determined by flow cytometry using Annexin V/PI staining (D), and anti‐EdU Alexa Fluor 488 staining (F). The data (B,D,F) is 1 representative experiment, and the graph then represents data from *n* = 5 replicate experiments (A,C,E). The data represent the mean ± S.E.M. of *n* = 5. *P < 0.05, **P <0.01, and ***P <0.001 compared with the control group. ^#^ *P* < 0.05 and ^###^ *P* < 0.001 compared with the LPS treatment group

We further examined whether the effects of NaSal on apoptosis and cell proliferation depended on activation of AMPK in LPS‐stimulated THP‐1 cells. For this, we used AICAR, an AMPK‐specific activator.^23^ We determined the total and phosphorylation levels of AMPK by Western blot analysis. As shown in Fig. 3A,B, treatment with AICAR reversed the LPS‐stimulated down‐regulation of p‐AMPK and the total protein level of AMPK had no obvious change. Apoptosis and cell proliferation of the THP‐1 cells was analyzed using Annexin V/PI staining and anti‐EdU Alexa Fluor 488 staining, respectively. In addition, we found that, AICAR similar to NaSal, significantly induced apoptosis from 9.52 ± 0.23% of the LPS‐stimulated THP‐1 cells to 25.95 ± 0.91% of the THP‐1 cells treated with LPS and AICAR (*P *< 0.001; Fig. 3C,D), and reduced the EdU incorporation rate to 26.69% (*P *< 0.001; Fig. 3E,F).

### 3.4 AMPK inhibition reverses the effects of NaSal on apoptosis and cell proliferation in LPS‐stimulated THP‐1 monocytes

**Figure 4:**
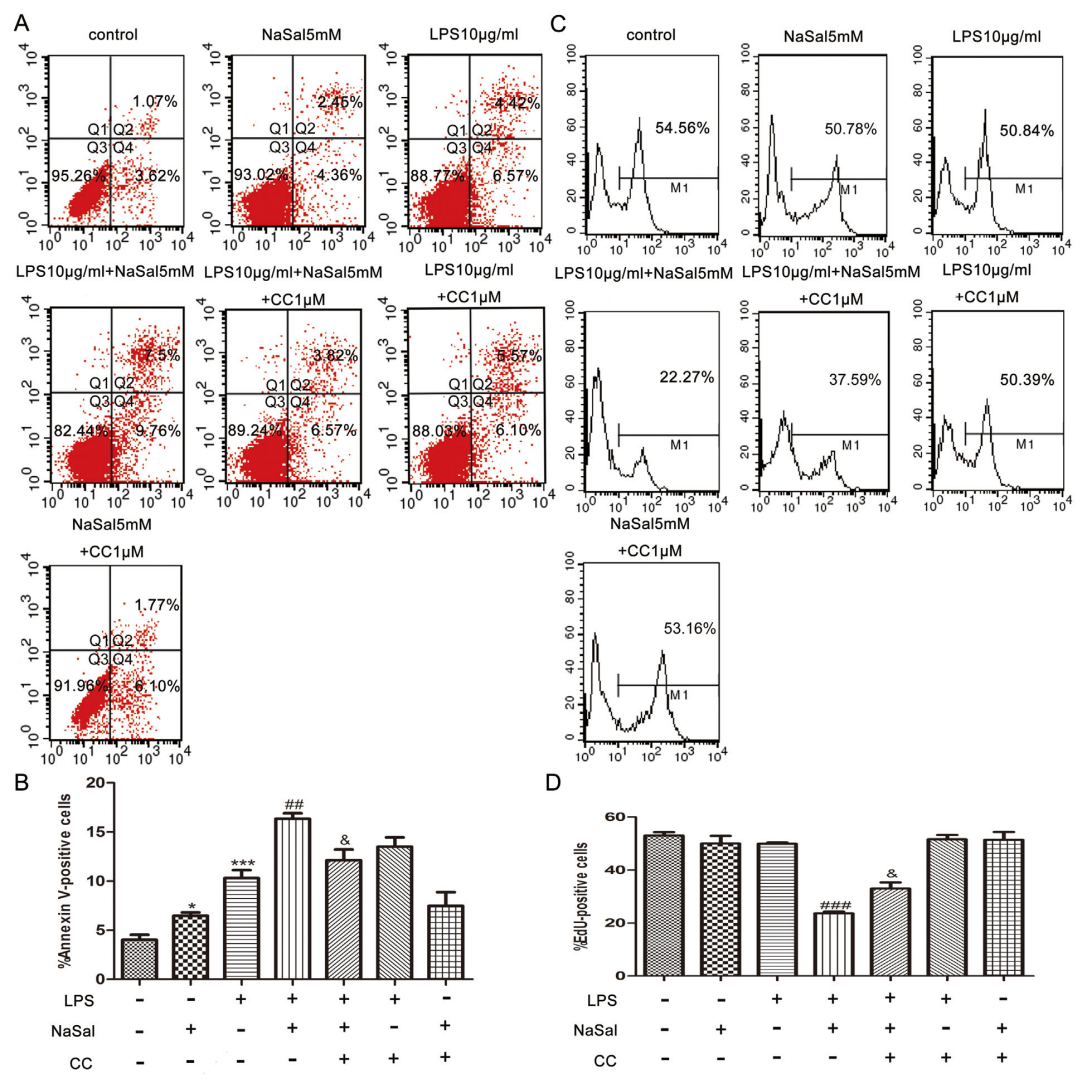
Blocking AMPK signaling inhibited the effects of NaSal on apoptosis and cell proliferation in LPS‐stimulated THP‐1 monocytes. Cells were pre‐incubated with Compound C (1 µM) for 1 h, then treated with LPS (10 μg/mL) for the next 24 h in the presence or absence of NaSal (5 mM). Apoptosis (B) and cell proliferation (D) were determined by flow cytometry using Annexin V/PI staining (A) and anti‐EdU Alexa Fluor 488 staining (C). The flow cytometry data (A,C) is 1 representative experiment, and the graph then represents data from *n* = 3 replicate experiments (B,D). The data represent the mean ± S.E.M. of *n* = 3. *P < 0.05 and ***P <0.001 compared with the control group. ^##^ *P* < 0.05 and ^###^ *P* < 0.001 compared with the LPS treatment group. ^&^ *P* < 0.05 compared with the LPS + NaSal treatment group

**Figure 5:**
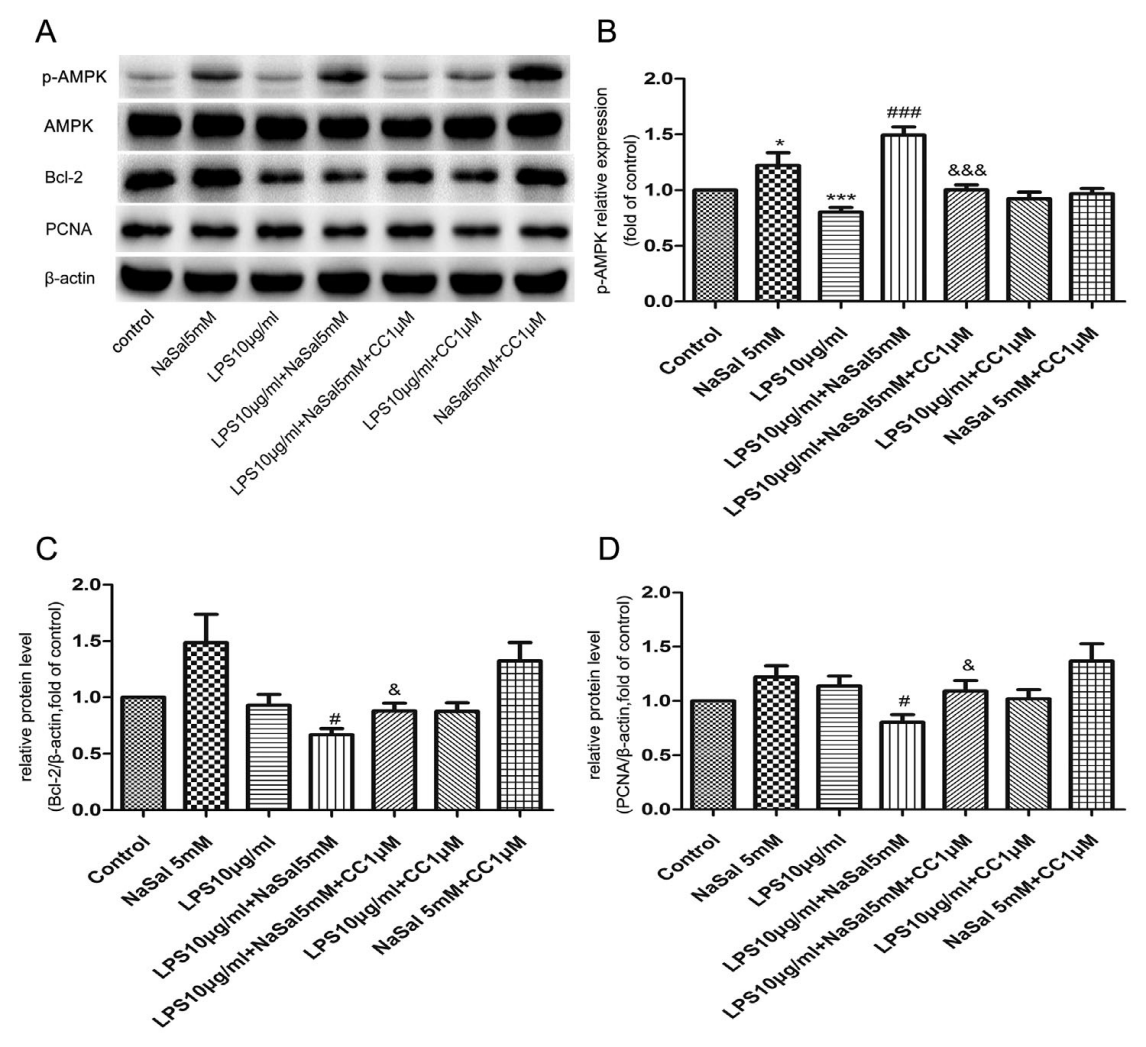
AMPK signaling pathway is involved in the pro‐apoptotic and anti‐proliferation effects of NaSal on LPS‐stimulated THP‐1 monocytes. Cells were pre‐incubated with Compound C (1 µM), then treated with LPS (10 μg/mL) for the next 24 h in the presence or absence of NaSal (5 mM). After incubation, cells were lysed and assessed by Western blotting using antibodies against p‐AMPK (Thr172), AMPK,Bcl‐2,PCNA, and β‐actin (A‐D). p‐AMPK, Bcl‐2, and PCNA protein expressions were assessed by Western blotting and densitometry. Values were normalized to AMPK‐1α or β‐actin expression and expressed as relative optical density. The relative expression of p‐AMPK, Bcl‐2, and PCNA in the control group was set at 1 for quantification. The Western blot data (A) is 1 representative experiment, and the graph then represents data from *n *> 3 replicate experiments (B‐D). The data represent the mean ± S.E.M. of *n *> 3. *P < 0.05 and ***P <0.001 compared with the control group. ^#^ *P* < 0.05 and ^###^ *P* < 0.001 compared with the LPS treatment group. ^&^ *P* < 0.05 and ^&&&^ *P* < 0.05 compared with the LPS + NaSal treatment group

To confirm the role of AMPK in NaSal‐mediated apoptosis and cell proliferation in LPS‐stimulated THP‐1 cells, we used Compound C (CC), an AMPK inhibitor, to suppress the cellular AMPK activity. As shown in Fig. 5A,B, compared with the LPS + NaSal group, pretreatment of the AMPK inhibitor CC (1 µM) suppressed the NaSal‐mediated AMPK activation in LPS‐stimulated THP‐1 cells. The total protein level of AMPK shows no obvious changes in different groups. We next examined cell apoptosis and proliferation by FACS analysis and Western blotting. Data from FACS analysis demonstrated that cells pretreated with Compound C reduced apoptosis but increased proliferation, when compared with the LPS + NaSal group (*P* < 0.05; Fig. 4B,D). The percentage of apoptotic cells decreased significantly from 16.34 ± 0.57% of the THP‐1 cells treated with LPS + NaSal to 12.13 ± 1.09% of the THP‐1 cells treated with LPS + NaSal + CC (*P* < 0.05; Fig. 4A,B). The EdU incorporation rate was increased to 37.59% in the cells treated with LPS + NaSal + CC compared with the cells treated with the LPS + NaSal (*P* < 0.05, Fig. 4C,D). There was no statistically significant difference between group NaSal and group NaSal + CC (or group LPS and group LPS + CC) (Fig. 4B,D). To further confirm the role of AMPK in NaSal‐mediated apoptosis and cell proliferation in LPS‐stimulated cells, we conducted another blocking experiment, in which cell proliferation and apoptosis were measured after AMPK was inhibited by using Western blotting. The expression of PCNA, a member of the DNA sliding clamp family of proteins that assist in DNA synthesis, is a well‐accepted marker of proliferation.^24^ The expression of Bcl‐2, an anti‐apoptotic protein that binds to its pro‐apoptotic relatives and neutralizes their pro‐apoptotic activity, can be used as an anti—apoptotic marker.^25^ Thus, we used Western blot analysis to investigate the effects of AMPK inhibition on apoptosis and cell proliferation by examining the expression of PCNA and Bcl‐2. As shown in Fig. 5A,C,D, compared with control cells, NaSal seems to increase Bcl‐2 and PCNA protein expression, but there was no statistical differences. However, the LPS + NaSal group decreased Bcl‐2 and PCNA protein expressions when compared with LPS‐treated cells. Cells pretreated with Compound C (1 µM) increased Bcl‐2 and PCNA protein expressions when compared with the LPS + NaSal group. In the presence of Compound C, the apoptosis induction and cell proliferation inhibition effects of NaSal were abrogated in LPS‐stimulated THP‐1 cells (Fig. 5C,D).There was no statistically significant difference between group NaSal and group NaSal + CC (or group LPS and group LPS + CC) in Bcl‐2 and PCNA protein expressions (Fig. 5C,D).

### 3.5 AMPK inhibition reverses the promotive effects of NaSal on TNF‐α, IL‐1β secretion in LPS‐stimulated THP‐1 monocytes

**Figure 6:**
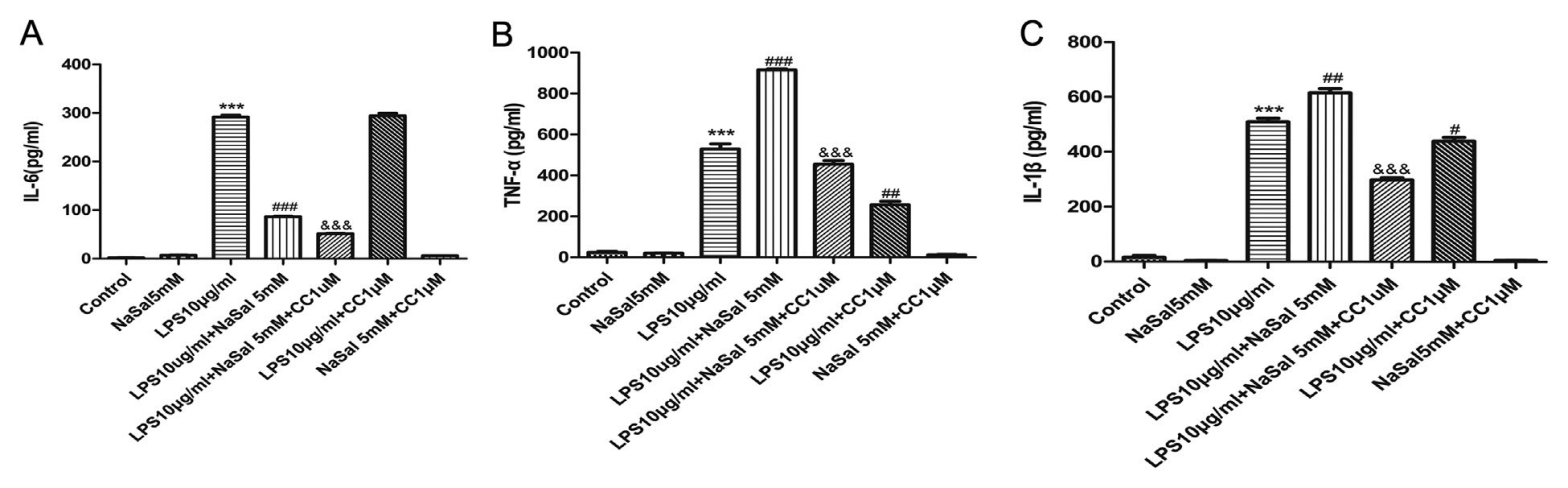
Effect of AMPK inhibition on LPS‐induced IL‐6, TNF‐α, and IL‐1β productions in the presence of NaSal. Cells were pre‐incubated with Compound C (1 µM), then treated with LPS (10 μg/mL) for the next 24 h in the presence or absence of NaSal (5 mM). Protein levels of IL‐6 (A), TNF‐α (B), and IL‐1β (C) in the culture supernatants were determined using ELISA assays. Values are expressed as the mean ± S.E.M. using results from three independent experiments. ***P < 0.001 compared with the control group. ^##^ *P* < 0.01 and ^###^ *P* < 0.001 compared with the LPS treatment group. ^&&&^ *P* < 0.001 compared with the LPS + NaSal treatment group

A recent study has shown that AMPK activation promotes IL‐6 production in cardiac fibroblasts.^26^ We examined whether AMPK activation is involved in the NaSal‐mediated pro‐inflammatory cytokines secretion. As shown in Fig. 6B,C, cells treated with NaSal increased LPS‐induced TNF‐α and IL‐1β secretions in THP‐1 monocytes. Moreover, compared with the LPS + NaSal group, inhibition of AMPK by Compound C completely abrogated the promotive effects of NaSal on LPS‐induced TNF‐α and IL‐1β productions. However, NaSal decreased LPS‐induced IL‐6 secretion and pretreatment of Compound C cannot change this inhibitory effect. There was no statistically significant difference between group NaSal and group NaSal + CC (or group LPS and group LPS + CC) in IL‐6 secretion (Fig. 6A). Also, the LPS + CC group reduced TNF‐α secretion and slightly inhibited IL‐1β secretion, when compared with the LPS‐treated cells (Fig. 6B,C) . Taken together, NaSal was shown to inhibit the LPS mediated activation of IL‐6, but enhance the effect on TNF‐α and IL‐1β. The effect of on TNF‐α and IL‐1β, but not IL‐6 was reversed by Compound C demonstrating that the TNF‐α/IL‐1β effect is AMPK‐mediated. AMPK activation is involved in NaSal‐mediated TNF‐α and IL‐1β secretion in LPS‐stimulated THP‐1 cells.

### 3.6 NaSal/AMPK activation inhibits STAT3 signaling activity

**Figure 7:**
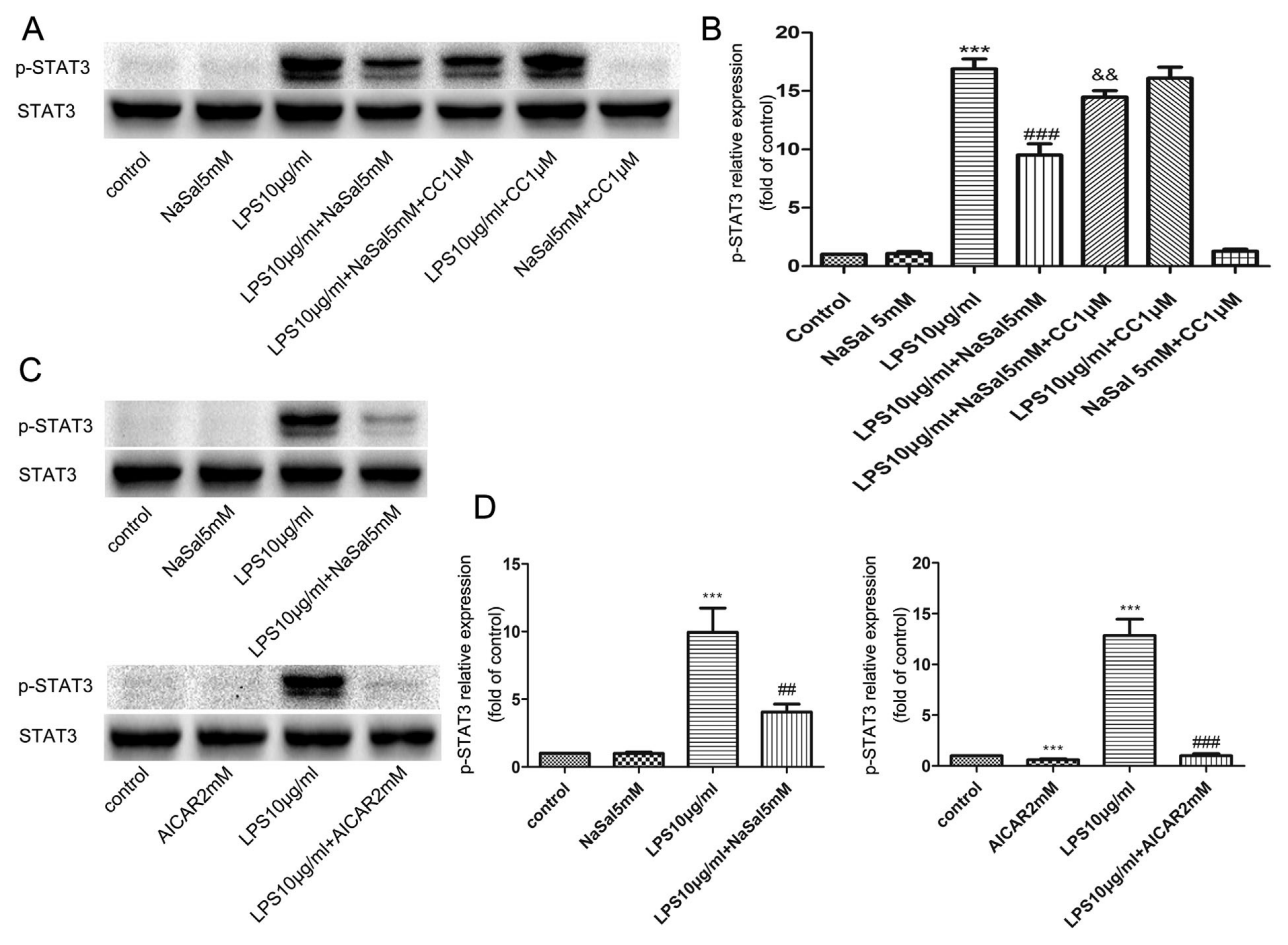
AMPK signaling pathway is involved in the inhibition of NaSal on LPS‐induced STAT3 phosphorylation. Cells were pre‐incubated with Compound C (1 µM), then treated with LPS (10 μg/mL) for the next 24 h in the presence or absence of NaSal (5 mM) or AICAR (2 mM). After incubation, cells were lysed and analyzed by Western blotting using antibodies against p‐STAT3 and STAT3. (A‐D) p‐STAT3 protein expression was assessed by Western blotting and densitometry. Values were normalized to STAT3 expression and expressed as relative optical density. The relative expression of p‐STAT3 in the control group was set at 1 for quantification. The Western blot data (A,C) is 1 representative experiment, and the graph then represents data from *n *> 3 replicate experiments (B,D). The data represent the mean ± S.E.M. of *n *> 3. *P < 0.05 and ***P < 0.001 compared with the control group. ^###^ *P *< 0.001 compared with the LPS treatment group. ^&&^ *P *< 0.001 compared with the LPS + NaSal treatment group

Because salicylate binds directly to AMPK, and STAT3 is critically involved in the regulation of inflammation, apoptosis, cell survival, and cell growth processes,^27^, ^28^ we further investigated whether AMPK activation by NaSal modulates STAT3 signaling activity. The total and phosphorylated levels of STAT3 in different groups were evaluated through Western blot analysis. As shown in Fig. 7A,B, NaSal effectively inhibited LPS‐induced STAT3 phosphorylation at Tyr705 and this inhibitory effect was reversed by AMPK inhibitor CC (1 µM). There was no statistically significant difference between group NaSal and group NaSal + CC (or group LPS and group LPS + CC) in p‐STAT3 protein expression (Fig. 7A,B). In different groups, the protein level of total STAT3 has no obvious change. Consistent with NaSal's inhibitory effect, activation of AMPK by AICAR abolished STAT3 phosphorylation when compared with the LPS group (Fig. 7C,D).

## 4 DISCUSSION

Salicylate is used for reducing pain, fever, inflammation, insulin resistance, and protecting against cancer. A recent report has proposed the idea that all these beneficial effects may not only include inhibition of cyclooxygenases and NF‐κB signaling, but also be partly mediated by AMPK activation.^15^ AMPK is a highly conserved sensor of cellular energy status that exists in essentially all eukaryotic cells.^29^ In response to energy stress, AMPK suppresses cell growth and biosynthetic processes directly or through its interaction with other signaling pathways. AMPK activation can regulate numerous processes, including cell growth, autophagy, apoptosis, and metabolism of inflammation. One aim of the present study is to evaluate the extent to which AMPK is involved in NaSal‐mediated inflammation in LPS‐stimulated THP‐1 monocytes. Apoptosis and cell proliferation are important parts in the process of inflammation and inflammation‐related diseases. A previous study has also shown that apoptotic cells protect mice against LPS‐induced shock by reducing pro‐inflammatory cytokines, suppressing neutrophil infiltration, and decreasing serum LPS levels.^30^ The results of flow cytometry testing and Western blot analysis in our study have shown that NaSal induced apoptosis, reduced proliferation, and down‐regulated the protein expressions of cell proliferating biomarker PCNA and anti‐apoptosis gene Bcl‐2 in LPS‐stimulated THP‐1 cells, while these effects could be partly reversed by the application of AMPK inhibitor CC. Our results found that NaSal inhibited LPS‐induced de‐phosphorylation of AMPK, promoted apoptosis and reduced cell proliferation in LPS‐stimulated THP‐1 monocytes and similar results were observed with AICAR, a distinct AMPK activator. NaSal and AICAR had an effect on apoptosis in both control and LPS‐treated cells, but the anti‐proliferative effect was only seen on LPS‐treated cells. Our study suggested that induction of apoptosis and inhibition of cell proliferation by NaSal in LPS‐stimulated THP‐1 cells is AMPK dependent. These results are in agreement with recent findings showing that AMPK is an apoptosis inducer and growth inhibitor due to metabolic stress, AMPK activation induced apoptosis, and reduced cell growth.^6^, ^12^ Activation of AMPK exerts cell growth inhibition and induction of apoptosis in human hepatocellular carcinoma,^23^ HT‐29 colon cancer cells,^6^ and in human bladder cancer T24 cells.^31^ However, AMPK has also been reported to be an anti‐apoptotic molecule and AMPK activation could block apoptosis in prevention of cell dysfunction associated with related diseases.^32^ Therefore, the effects of apoptosis induction and cell growth inhibition through AMPK activation might be dependent on cell types and experimental condition.

AMPK as a suppressor of inflammation has been proposed.^33^ AMPK activation inhibited the LPS‐induced expression of pro‐inflammatory cytokines in macrophages,^34^ BV2 microglia cells,^35^ and mesangial cells.^36^ However, recent studies have also suggested pro‐inflammatory effects of AMPK by demonstrating that AMPK activation increases the secretion of cytokines, including IL‐1β and IL‐6.^26^, ^37^ AMPK activation may have the opposite biological effects on cytokine release in different cell types. Monocytes are an important part of the immune system and respond to LPS partly by expressing inflammation‐related cytokines (IL‐1b, IL‐6, IL‐8, IL‐10, and TNF‐a).^38^ We thus examined the role of AMPK activated by NaSal in LPS‐stimulated THP‐1 monocytes, and found that NaSal promoted TNF‐α and IL‐1β but inhibited IL‐6 secretions in LPS‐stimulated THP‐1 monocytes. To verify the role of AMPK in NaSal‐mediated inflammation‐related cytokines secretion in THP‐1 cells, we blocked AMPK activity via Compound C and found that NaSal induced TNF‐α and IL‐1β secretion in an AMPK‐dependent manner but inhibited IL‐6 secretion in an AMPK‐independent manner. AMPK activation by NaSal also has a pro‐inflammatory effect in LPS‐stimulated THP‐1 cells by increasing the production of TNF‐α and IL‐1β. In our view, inhibition of the upstream activator of AMPK might be involved in NaSal‐mediated IL‐6 secretion in LPS‐stimulated THP‐1 cell. The Ca^2+^/calmodulin‐dependent kinase kinases (CaMKKs) is an upstream activator of AMPK,^39^ and a previous study demonstrated that IL‐6 is AMPK independent and is mediated via a Ca^2+^‐dependent pathway in skeletal muscle cells.^40^ A previous study has shown that AMPKα1 activation can activate the NF‐κB system and inhibition of NF‐κB abolished the effects of AMPK activation on protein expressions.^41^ We guess that AMPK activation of NF‐κB signaling might participate in NaSal‐induced TNF‐α and IL‐1β secretions in our work. A recent study has reported the beneficial effects of NaSal in numerous experimental models of sepsis by triggering lipoxins, blocking microbial mediators of sepsis, reducing NF‐κB stimulation, and inhibiting septic pathways.^42^ The different effects may account for the different findings from models with nonsteroidal anti‐inflammatory drug (NSAID) intervention and further study is required to investigate the potential role of AMPK in NaSal‐mediated sepsis.

STAT3 plays a vital role in regulating the expression of various downstream genes that control stress response, immune function, inflammation, and metastasis of cancer. STAT3 participates in inflammation‐associated diseases through regulating cell proliferation and inducing the expression of anti‐apoptotic genes, such as Bcl‐2 and Bcl‐xl.^20^, ^43^ However, the relationship between STAT3 and AMPK under the inflammation condition after NaSal treatment is not well understood. In our study, we stimulated THP‐1 cells with LPS in the presence or absence of AMPK activators NaSal and AICAR, employed AMPK inhibitor Compound C, and measured the phosphorylation statuses of AMPK and STAT3 proteins. Here, we observed AMPK activation was accompanied by STAT3 inactivation, and STAT3 inactivation was reversed by the application of Compound C. Therefore, our results indicated that activation of AMPK by NaSal inhibited STAT3 signaling activity. A previous study demonstrated that AMPK activation by metformin inhibited STAT3 signaling, and thus inhibited HCC cells growth in vitro and in vivo.^23^ Another study has also reported that metformin, an AMPK activator, induced growth inhibition through inactivation of Stat3‐Bcl‐2 pathway in ESCC tumor cells.^44^ However, whether the AMPK‐STAT3 axis participates in NaSal‐mediated apoptosis, cell proliferation, and inflammatory cytokines is still unknown in our work, and further study is warranted.
